# The effects of health care programs for gestational diabetes mellitus in South Korea: a systematic review

**DOI:** 10.4069/kjwhn.2020.10.28

**Published:** 2020-12-08

**Authors:** Seo Jin Park, Jina Lee

**Affiliations:** 1 Department of Nursing, Donggang University, Gwangju, Korea; 2 Christian College of Nursing, Gwangju, Korea

**Keywords:** Diabetes mellitus, Gestational diabetes, Health, Program, Systematic review

## Abstract

**Purpose:**

The purpose of this study was to investigate the effects and characteristics of health care programs for pregnant women with gestational diabetes mellitus (GDM) in Korea.

**Methods:**

This study was conducted according to the Cochrane Collaboration’s systematic literature review handbook and the Preferred Reporting Items for Systematic Reviews and Meta-Analyses reporting guideline. We searched eight international and domestic electronic databases for relevant studies. Two reviewers independently selected the studies and extracted data. For each study, information on the research method, participants, characteristics of the program, and results were extracted using a previously established coding table. The National Evidence-based Healthcare Collaborating Agency’s risk of bias assessment tool for non-randomized studies was used to assess the risk of bias of the included articles. A qualitative review of the selected studies was performed because the interventions differed considerably and the measured outcomes varied.

**Results:**

Out of 128 initially identified papers, seven were included in the final analysis. The risk of bias was evaluated as generally low. Health care programs for pregnant women with GDM showed positive effects on blood glucose control. Anxiety and depression were reduced, and self-management and self-care behavior, self-efficacy, and maternal identity improved.

**Conclusion:**

Our study provides clinical evidence for the effectiveness of health care programs for pregnant women with GDM, and its results can be used to support the development of health care programs for GDM. More well-designed research is needed on GDM, especially studies that deal with emotional stress and apply a family-oriented approach.

## Introduction

Gestational diabetes mellitus (GDM) is an important health care issue that occurs in one in six pregnant women worldwide [1]. In the past, GDM was defined as occurring at any point in pregnancy, regardless of the extent of the disease. Recently, however, the American Diabetes Association (ADA) has clearly defined GDM as diabetes diagnosed in the second or third trimester of pregnancy [2]. According to data from the Korean Diabetes Association in 2013, the prevalence of GDM in Korea increased from 4.1% in 2007 to 10.5% in 2011 [3], and the incidence of GDM relative to the number of babies born in 2017 was estimated to be 15.3% [4,5].

The risk factors for GDM include heredity (family history) and environmental factors (age, obesity, high-fat diet, etc.) [6], and the prevalence of GDM is expected to further increase gradually due to the increasing age of childbirth and changes towards westernized eating habits among Korean women. GDM may recur in 33% to 50% of subsequent pregnancies [7], and GDM is associated with a high risk of type 2 diabetes, a lifelong condition [8]. Above all, GDM has a serious impact on the mother and fetus [2], including elevated risks of premature birth and maternal overweight, preeclampsia, cesarean section, fetal macrosomia, and trauma during delivery. After delivery, the child also has increased risks of disability due to obstetric complications, hypoglycemia, hypocalcemia, hyperbilirubinemia, respiratory distress syndrome, and obesity [8].

Early detection is important to improve the prognosis of GDM and to reduce the risk of health-related problems in the mother and fetus, and careful health management for pregnant women is required to maintain appropriate blood glucose levels [8,9]. In particular, in GDM, blood glucose levels can be managed only by diet and lifestyle modifications [1]; therefore, for management of blood glucose in pregnant women to be successful, patients themselves must have a solid knowledge of the disease and perform self-care. Consequently, health education and training are important for continuous blood glucose management [10]. However, pregnant women who are found to have GDM may not know much about the disease or blood glucose management because they have not experienced the disease before [10], and most pregnant women experience anxiety, depression, fear, and stress about responding to health problems that may negatively affect the fetus [11]. The adaptations required to manage GDM, in addition to the physiological and psychological changes of pregnancy, cause additional stress regarding blood glucose control and disease burden, which can reduce the effectiveness of treatment [12]. GDM may also adversely affect the health care behaviors of pregnant women and the formation of maternal identity through complex factors [10]. The care goals for women with GDM are aimed at preventing complications in the mother and fetus based on early detection and treatment, with the ultimate objective of safe birth [10]. Therefore, the control of blood glucose levels in women with GDM is paramount, and health care providers need to provide comprehensive health care interventions tailored to both the physiological changes and the individual needs of pregnant women [9-11]. For clinicians caring for Korean women with GDM, it would thus be beneficial to conduct a systematic analysis of health care programs (education, intervention methods, etc.) implemented for Korean women with GDM, with an analysis of their effectiveness, methods, and content. However, no such study has yet been carried out, despite the steady increase of GDM in Korea.

### Purpose of research

The purpose of this study was to systematically review studies of health care programs conducted among Korean women with GDM by examining the general characteristics of the selected studies and analyzing the effectiveness of the health care programs described therein.

## Methods

Ethics statement: This study is a literature review of previously published studies and was therefore exempted from research approval by the Institutional Review Board of Christian College of Nursing (No. CCN-2019-5).

### Research design

This study is a systematic review of the effects of health care programs for pregnant Korean women with GDM.

### Criteria for selection and exclusion of studies

This review was conducted in accordance with the systematic reviews handbook [13] of the Cochrane Collaboration and the systematic review guidelines of the Preferred Reporting Items for Systematic Review and Meta-Analysis (PRISMA) [14]. First, the researchers identified the PICO-SD (participants, intervention, comparison, outcomes, and study design) parameters, and then searched electronic literature databases according to the following processes for selection and exclusion.

#### Selection criteria

##### (1) Participants (P)

This study targeted Korean women with GDM, who had not been diagnosed with diabetes before pregnancy and were diagnosed with GDM by doctors between 24 and 28 weeks of pregnancy. No limitations were placed on the number of pregnancies and age at pregnancy.

##### (2) Intervention (I)

The interventions were health care programs that included physical and/or psychosocial health management for pregnant Korean women with GDM. The main literature search strategy included all types of programs involving education, counseling, online health care interventions, and so forth.

##### (3) Comparison (C)

The comparison groups in this study were GDM pregnant women who were provided with no intervention or general diabetes interventions (drugs, diet, and exercise) that did not include the interventions applied to the experimental group.

##### (4) Outcomes (O)

The preliminary literature review indicated that a wide variety of outcome variables were reported; therefore, this study did not limit the outcome variables in the literature selection criteria.

##### (5) Study design (SD)

Studies that employed randomized and non-randomized experimental studies with controls were included in this review.

#### Exclusion criteria

The exclusion criteria for selecting studies were: (1) studies with participants who were not Korean pregnant women (e.g., marriage migrant women, foreigners, etc.); (2) studies with a research design beyond our purpose (e.g., single-group experimental studies, policy studies, survey studies, qualitative studies, systematic research, etc.); (3) studies that did not report the effectiveness of intervention programs; (4) studies of delivery methods; and (5) studies not written in English.

#### Searching and selecting literature

##### (1) Searching literature

Prior to the literature search, keywords for each electronic literary database were selected based on the PICO-SD, and the search strategies utilized both MeSH phrases (MeSH Descriptor Data 2018) and unstructured natural language terms. The international databases used in this study were Ovid MEDLINE, the Cochrane Central Register of Controlled Trials (CENTRAL), Cumulative Index to Nursing and Allied Health Literature (CINAHL), and ProQuest. As Korean databases, KoreaMed, Research Information Sharing Service (RISS), Korean Studies Information Service System (KISS), and DBpia were also used. The reference lists of the selected studies were also manually searched. The keywords used for the search were [‘Diabetes, Gestational’ (MeSH)] and [‘Korean’ (MeSH)] and [‘Intervention’ or ‘Program’ or ‘Education’ (MeSH)] in the databases. If a dissertation was published in an academic journal, only the published version was included, and if the same study by the same author was found in both Korean and international databases, only the Korean version was included. The final literature search date for this study was July 14, 2019.

##### (2) Selection of literature

The literature retrieved through the database search was compiled using EndNote® (EndNote X8, Thomson Reuters, New York, NY, USA) and Microsoft Excel 2016 (Microsoft, Redmond, WA, USA). In the first stage of the selection process, studies were included or excluded based on a title and abstract review, while in the second stage of the selection process, the original text of the studies selected in the first step was examined to make the final choice regarding inclusion. For the final seven selected studies, research methods, subjects, and characteristics and results of the exercise intervention were extracted using a pre-established coding scheme. The entire process of selection and data extraction was carried out independently by the two researchers, and in cases of disagreement, the original text was reviewed and consensus was reached. Kappa analysis was performed using IBM SPSS ver. 23.0 (IBM Corp., Armonk, NY, USA) to confirm agreement between the researchers in the literature selection process.

##### (3) Quality evaluation of the literature

All of the studies analyzed herein were non-randomized control experimental studies, and the risk of bias assessment tool for non-randomized studies (RoBANS) developed by the National Evidence-based Healthcare Collaborating Agency [15] was used to evaluate the quality of the studies. The RoBANS consists of six items in total: selection of participants, confounding variables, measurement of intervention (exposure), blinding for outcome assessment, incomplete outcome data, and selective outcome reporting. Depending on what a study describes, the risk of bias is assessed as low, high, or uncertain. Both researchers independently assessed the quality of the studies, discussed any items about which they initially disagreed, and reached consensus after a joint reassessment process.

### Method of data analysis

Due to the diverse outcomes of the health care programs, this study analyzed the effects in the following domains: physiological, cognitive, behavioral, and psychosocial effects.

## Results

### Results of literature selection

**Figure. 1.**
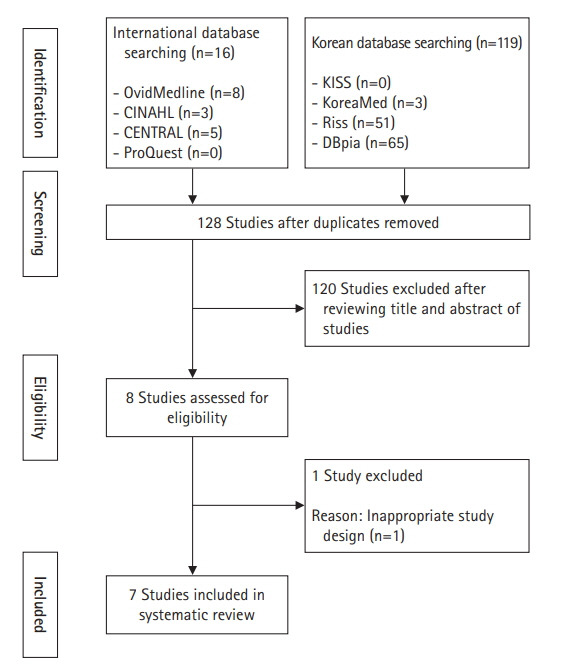
Flow diagram of study selection.

In total, 119 documents were identified through the domestic electronic literature databases (KISS, KoreaMed, RISS, and DBpia) and 16 documents through the international electronic literature databases (Ovid MEDLINE, CINAHL, CENTRAL, and ProQuest). After the exclusion of seven duplicates, 128 studies were initially reviewed based on their title and abstract. This step yielded eight documents; after full-text review, one study that only involved a single group was excluded, resulting in seven studies [9,16-21]. The reference lists of these seven studies were manually reviewed, but no further studies were included (Figure 1). The concordance between the two researchers in selecting the literature was fairly high (kappa=0.81; *p*<.001) [22].

### General characteristics of the studies

The seven finally selected articles were all (100%) non-randomized control experimental studies, similar to the pre-post nonequivalent quasi-experimental design. Four studies (57.1%) were published in journals [9,16-18], and the others were two of unpublished doctoral dissertations (28.6%) [19,20] and a master’s thesis (14.3%) [21]. One study was published in 2001 [21], and the remaining six were published from 2013 to 2018. Four studies (57.1%) delivered offline programs [9,16,18,21], one study (14.3%) described an online program [19], and two studies (28.6%) used both modalities [17,20]. The programs in four studies (57.1%) were individual-based [16,17,19,20], while one study (14.3%) utilized a small-group design [21] and two studies (28.6%) combined individual and small-group interventions [9,18]. The study subjects were all pregnant women diagnosed with GDM between 24 and 28 weeks of pregnancy. In six studies (85.7%), the program was provided during pregnancy, while one study (14.3%) [17] provided a postpartum program. All seven programs (100%) were conducted in hospitals. The average age of subjects ranged from 31.5 to 35.5 years in the experimental group and from 31.8 to 36.4 years in the control group, although one study [18] did not describe participants’ age. The programs were delivered 4 to 16 times, and the time required per session ranged from at least 5 minutes [17] to a maximum of 60 minutes [18]. All studies reported pregnancy outcomes, and two (28.6%) also reported newborn outcomes [20,21] (Table 1).

The most common physiological outcome variable was the level of blood glucose (n=6, 85.7%) in pregnancy, followed by gestational age at delivery and delivery mode (n=2, 28.6%). The two studies (28.6%) reporting newborn outcomes presented data on weight [20,21] and neonatal complications at birth [20,21]. Five studies (71.4%) measured psychosocial outcome variables, with the most common being depression (n=3, 42.9%) [16,18,29], followed by self-efficacy [16,17], anxiety [16,19] (each n=2, 28.6%,), and maternal identity (n=1, 14.3%) [9]. The psychosocial outcome variables were all measured using structured questionnaires. Of the four studies that reported behavioral outcome variables, self-management [9,17] and self-care behaviors [18,19] accounted for two (28.6%) each. Finally, outcome variables in the cognitive domain were evaluated in only one study (14.3%), which measured knowledge about GDM [18] (Table 2). Regarding the content of the interventions, self-care was most common (n=2, 28.6%) [9,19], followed by one study (14.3%) each on diet [21], lifestyle improvement [18], exercise [20], and postpartum care [17]. The details of the programs are presented in Table 1.

### Quality evaluation

The quality assessment by RoBANS [9,16-21] found that all seven studies (100%) were assessed as having a low risk of bias due to satisfactory selection of the target group. Regarding selection bias caused by an inadequate identification of confounders, one study (14.3%) [21] was found to have a high risk of bias due to a failure to identify the major confounding variable, while the remaining studies (n=6, 85.7%) adequately identified the major confounding variables and controlled for them in the analysis. The risk of bias due to inappropriate intervention (exposure) measurements was evaluated as low because all studies used either physiological measurements or structured questionnaires. For blinding to outcome evaluation, one study (14.3%) [18] noted that a third researcher collected data, whereas three (42.9%) [17,20,21] did not use blinding, but this did not affect the results; thus, those studies were determined to have a low risk of bias. For three studies (42.9%) [9,16,19], however, the risk of bias was judged to be high because the blinding was incomplete and could possibly have affected the results. Regarding attrition bias caused by the improper handling of incomplete data, two studies (28.6%) [17,18] were determined to be low-risk as they reported losses of less than 20%, while four studies (57.1%) [9,16,19,20] were evaluated as being high-risk because the attrition rate was higher than 20%. One study (14.3%) [21] was determined to have uncertain risk in this domain due to the lack of a description of the attrition rate. Reporting bias was determined to be low-risk because all studies reported results based on the expected variables, which were planned in advance.

**Figure. 2.**
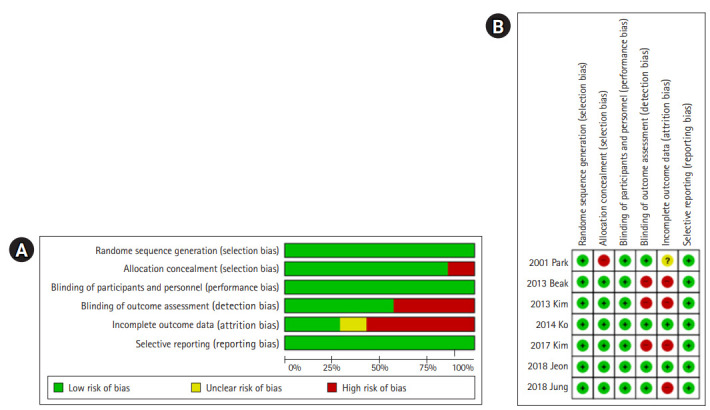
Risk of bias. (A) Risk of bias summary. (B) Risk of bias for selected studies.

Given the above results, the overall risk of bias of the seven studies was assessed as low (Figure 2).

### Effects of the health care programs

Six of the seven studies included in the systematic literature review reported that the program was effective for the physiological health of the pregnant women, while one study [21] was not able to determine the exact effectiveness of the program because no *p*-value was reported. Five studies (71.4%) [9,17-20] presented physiological measurements related to glucose metabolism, including 2-hour post prandial blood glucose levels (PP2, n=2), glycated hemoglobin levels (HbA1c, n=3), fasting blood sugar levels (FBS, n=3), oral glucose tolerance testing (OGTT, n=2), 1-hour post prandial blood glucose levels (PP1, n=1), and glycated albumin levels (n=1). The studies reporting HbA1c [18,19] showed significant reductions in the experimental group (t=3.94, *p*<.001 and F=31.22, *p*=.001), as did the studies reporting PP2 [9] (U=–2.43, *p*=.015) and FBS [18] (t=5.03, *p*<.001). However, for OGTT [17,20], PP1 [19], and glycated albumin [19], no significant differences were found between the experimental and control groups. Of the two studies that reported newborn outcomes, one study [21] did not provide the *p*-value and the effectiveness therefore could not be determined. In Jung’s study [20], no significant differences were found for birth weight, macrosomia, Apgar scores, hypoglycemia, or trauma at birth.

In the psychosocial domain, depression was measured in three studies (42.9%) [16,18,19], of which two [16,18] reported a statistically significant decrease in the experimental group (t=3.53, *p*=.001 and F=4.27, *p*=.043, respectively). Anxiety was evaluated in two studies [16,19] and both studies reported statistically significant reductions (t=5.49, *p*<.001 and F=4.13, *p*=.048, respectively). Self-efficacy also showed significant results in the two studies that reported corresponding measurements [16,17] (t=–2.06, *p*=.047 and t=–2.02, *p*=.048, respectively). The study that assessed maternal identity [9] also showed significant positive results for the experimental group (U=–4.48, *p*<.001).

In the behavioral realm, self-management and self-care behaviors were reported in four studies (57.1%) [9,17-19], with statistically significant improvements in the experimental group in one study each [9,18] (U=–3.80, *p*<.001 and t=–3.25, *p*=.002, respectively). However, the level of GDM knowledge, measured as a cognitive outcome [18], did not show a significant difference (t=–1.98, *p*=.052) (Table 1).

## Discussion

This systematic review examined the effects of seven studies that described health care programs provided to Korean pregnant women with GDM and reported the physiological, behavioral, cognitive, and psychosocial effects of those programs.

All of the studies were non-randomized experimental studies, and the majority (n=6) were carried out since 2013. More broadly, intervention studies including medication, diet, and exercise therapy have been carried out for women with GDM from diverse linguistic and cultural backgrounds [23,24]. The growing prevalence of GDM in Korea, related to the aging of pregnant women, seems to have contributed to an increasing interest in GDM [20]. In the studies analyzed herein, the health care programs tended to be provided on an individual basis (n=4), rather than in small groups, and through offline delivery (n=4), although three studies used online [19] or combined online and offline modalities [17,20]. Although a prior study [11] suggested that the effects of programs might differ according to the delivery method, this study could not clearly identify any such effects due to an insufficient number of studies. Various intervention strategies and methods, including case management and information technology-based programs, were used in the research analyzed herein, and five of the seven studies included physical and lifestyle interventions, such as self-measurement of blood glucose levels, diet, and exercise. This is thought to reflect the importance of diet and exercise as ways to improve blood glucose levels in patients with GDM; exercise and diet are major management methods for GDM, just as they are for type 1 and type 2 diabetes [20], especially since insulin alone does not provide sufficient blood glucose control in GDM. Although the effect size of the intervention method could not be determined due to the heterogeneity of the studies, researchers should investigate interventions and approaches that reflect the needs of women with GDM, considering that GDM, which is diagnosed at 24-28 weeks of pregnancy, requires regular self-care, both during pregnancy and after childbirth [11].

Most of the selected studies (n=6) confirmed blood glucose control as a physiological outcome. The reported parameters included HbA1c, FBS, OGTT, post prandial blood sugar, and glycated albumin, and two or more physiological indicators were analyzed in four studies. The ADA and the World Health Organization recommend monitoring HbA1c, as it reflects the average blood glucose level within 3 months and serves both as a diagnostic criterion for diabetes and as an indicator of blood glucose control [25,26]. Although HbA1c is generally a reliable indicator, it may be affected by the physiological diabetogenic effects of pregnancy, so appropriate blood sugar testing needs to be performed starting on the first prenatal visit when GDM risk is suspected [1]. Glycated albumin, which reflects changes in blood sugar within weeks due to the shorter half-life of albumin relative to hemoglobin, has the advantage of detecting changes in blood glucose control over relatively short intervals compared to glycated hemoglobin; in particular, it sensitively reflects post prandial blood glucose [26]. GDM requires more stringent blood glucose control goals than type 1 or 2 diabetes [27], since macrosomia, the main complication of GDM, is primarily related to post prandial hyperglycemia [28]. Despite rigorous attempts to control blood glucose levels based on glycated albumin measurements [26] and the lack of evidence that one test method is superior to the other [29], the complications of diabetes may progress during pregnancy. One study analyzed herein focused on the postpartum period. GDM pregnancies are considered high-risk, and women with GDM are also at an elevated likelihood of developing diabetes in the future, which underscores the importance of regular blood glucose tests to prevent diabetes after delivery [29]. Although no consensus has been reached yet on when and how to detect postpartum abnormalities in women diagnosed with GDM [29], the ADA recommends a 75-g OGTT at 4 to 12 weeks after delivery, and every 1 to 2 years afterward [1,2]. As such, thorough postpartum care, including blood glucose management, is important for women with GDM [2,8].

There were only two studies each that reported self-care and self-management as behavioral outcome variables. An integrated self-care program, a comprehensive lifestyle-modification coaching program, and a web-based self-care program were effective for blood sugar control for among pregnant women with GDM. Since improvements in diet and exercise play a more foundational role in treating GDM than is the case for other types of diabetes, it is important to promote self-care to encourage women to actively seek out lifestyle modifications [18]. However, many women with GDM have reported that self-care in terms of changing diet and exercise was difficult [17]. Considering that a lack of lifestyle improvements after childbirth often leads to type 2 diabetes [1,16], developing health care programs that can encourage sustained self-care in terms of lifestyle improvement is important.

The single study that reported a cognitive outcome variable did not find improvement in GDM knowledge. This is possibly related to the fact that the program focused on coaching to improve self-care capability for blood glucose control rather than education. Pregnant women with GDM, in particular, have been reported to have low levels of knowledge regarding weight management, hypoglycemia treatment, and exercise methods for blood glucose control [10]. Although improvement of knowledge does not always lead to positive behavioral changes, it is necessary to consider strategies that can improve specific knowledge when developing programs to promote self-care targeting lifestyle modifications.

While the psychosocial effects of the programs varied, including depression (n=3), anxiety (n-2), self-efficacy (n=2), and maternal identity (n=1), the number of studies was limited, making it difficult to present quantitative estimates of intervention effects. As psychosocial difficulties can have a negative effect on blood glucose management by reducing the treatment effect [12], assessing psychosocial outcomes is important for pregnant women with GDM. Pregnant women with GDM have been reported to experience greater psychological anxiety and depression due to higher physical and psychological fatigue than their healthy counterparts [30], concerns about maternal and fetal effects of GDM [10], and guilt [11]. Thus, pregnant women with GDM not only have educational needs for blood glucose management but also require emotional support to reduce the anxiety and stress they feel [10,11]. However, current care for GDM mainly tends to focus on checking fetal health and the physical and hormonal changes in pregnant women, often overlooking the psychological care needs of women with GDM [16]. This review supports the need for more psychosocial interventions to promote acceptance, coping, and adaptation to GDM.

Family members or spouses did not participate in any of the interventions. However, family support as perceived by the pregnant women with GDM was important, and active support from family members was a factor associated with success for diet changes and self-care behavior in pregnant women [11]. If the family neglects GDM or blames it on the pregnant woman, the pregnant woman may feel guilt and stress, subsequently becoming less motivated to manage her health [12]. As the diagnosis of GDM may cause a sense of being overwhelmed, increasing women’s knowledge of GDM and ensuring cooperation in managing their health can enable pregnant women with GDM to recognize their current situation in a more positive light and to maintain stable diabetes management [12,30]. It would be beneficial for future interventions to engage family members as well [11].

The quality of the studies included in this study was assessed as high, considering the overall low risk of bias. The number of selected studies, however, was small and all of them were non-randomized control experiment studies. Given the lack of randomized studies, the scope for generalizing and interpreting the mediating effects presented by the selected studies is limited.

Nonetheless, this review contributes to the body of knowledge on GDM by reviewing and presenting the effects of health care programs, identifying the current situation of interventional research conducted to date, and confirming the methods, content, and effects of interventions. Future studies should attempt to use a randomized controlled trial design, and meta-analyses should be conducted to clarify the clinical effects. Individual education should also be provided to identify and implement mental health-related programs that reduce negative emotions and stress, such as anxiety and depression, and the development of programs with family-oriented approaches and a focus on the educational needs for health care for pregnant women with GDM should be prioritized.

## Figures and Tables

**Table 1.** Characteristics of the eligible studies (N=7)

First author (year)	Description of the program	Study size		Content of program		Format/target	Duration of curriculum
		Exp	Con	Exp	Con		
Jung (2018) [20]	IT-based exercise education and monitoring	49	127	· IT-based exercise education consisting of theoretical education, exercise program delivery, exercise journal evaluation, expert coaching, and online counseling and monitoring	Usual care about blood glucose management, nutrition, and exercise	Online+offline/individual	· 1 time/wk
							· 16 wk
Jeon (2018) [17]	Postnatal care program	30	32	· The program was composed of education and telephone counseling based on the Health Belief Model	None	Online+offline/individual	· 20 min×1 time+5 min×3 times
				· Booklets, video training: importance of postpartum care for pregnant women with GDM, timing and method of follow-up after delivery, and health management to prevent diabetes after delivery			· 12 wk
				·Phone consultation: consultation on overall health after delivery, diet, exercise and weight management, breastfeeding, stress management, and support			
Kim (2017) [19]	Web-based self-management program	22	22	· Web-based program including blood sugar management, diet therapy, physical activity, weight management program	General diabetes diet education	Online/individual	· 20–30 min×12 times
				· Consisted of counseling, emotional support, and education			· 12 wk
Ko (2014) [18]	Coaching program on comprehensive lifestyle modification	34	34	· The program was composed of counseling, encouragement, and support by GROW coaching	Usual care using a diabetes education brochure	Offline/small group and individual	· 30–60 min × 4 times
				· The content of the program consisted of diet, exercise, and self-blood glucose measurement			· 4 wk
				· Consisted of training (30 min), small-group coaching (30 min), and telephone coaching (20 min)			
Kim (2013) [9]	Integrated self-management program	28	27	· The program was composed of emotional support, education, decision-making control for self-management, blood glucose management, and maternal identity improvement	Usual care	Offline/small group and individual	· 1 hr×3 times+10–15 min×2 times
				· 5 times in total, three small-group meetings (1 hr each), and two phone calls for 10–15 min			· 5 wk
Baek (2013) [16]	Case management program	19	18	· Face-to-face interview: the same individual training as the one control group	Individual education and diabetes education brochure (blood glucose management, exercise therapy, weight management, postpartum care, caring for one’s body when sick, diabetes complications, and diet)	Offline/individual	· 5 times
				· Telephone interviews (5 times/2 wk): case management program for reassessment, diagnosis, performance, and evaluation of nursing needs			· 2 wk
Park (2001) [21]	Diet therapy	26	25	· Carbohydrate-restricted diet: the energy ratio of total calories was 45% carbohydrates, 20% protein, and 35% fat	· Normal carbohydrate diet: the energy ratio of total calories was 60% carbohydrates, 20% protein, and 20% fat	Offline/small group	Not described

Con: control group, Exp: experimental group, GROW: goal-reality-options-will, GDM: gestational diabetes mellitus, IT: information technology.

**Table 2.** Summary of the effects of the programs (N=7)

First author (year)	Physiological outcomes		Psychosocial outcomes			Behavioral outcomes	Cognitive outcomes
	Pregnancy outcomes	Infant outcomes	Depression/anxiety	Self-efficacy	Maternal identity	Self-management/Self-care behavior	Knowledge
Jung (2018) [20]	· Glycemic control	· Infant birth weight: not significant (*p*=.854)	-	-	-	-	-
	-Experience of insulin treatments: not significant (*p*=.657)	· Occurrence of macrosomia: not significant (*p*=.399).					
	-OGTT: not significant (*p*=.943)	· Apgar score: not significant (*p*=.376)					
	-SMBG (FBS/PP2): not significant (*p*=.246/*p*=943)	· Hypoglycemia occurrence: not significant (*p*=.405)					
	· Length of gestation: not significant (*p*=.899)	· Trauma at delivery: not significant (*p*=.263)					
	· Delivery type: not significant (*p*=.388)						
	· Morbidity of T2DM after delivery: not significant (*p*=.764)						
Jeon (2018) [17]	· Glycemic control:	-	-	· Self-efficacy: increased in the Exp (t=–2.02, *p*=.048)	-	· Self-management: not significant (t=–1.28, *p*=.206)	-
	-75 g oral glucose tolerance test: not significant (t=0.11, *p*=.748)						
Kim (2017) [19]	· Glycemic control	-	· Depression: not significant (F=2.90, *p*=.096)	-	-	· Self-care behavior: not significant (F=0.78, *p*=.381)	-
	-HbA1c: reduced in the Exp (F=31.22, *p*=.001)		· Anxiety: reduced in the Exp (F=4.13, *p*=.048)				
	-Glycated albumin: not significant (F=0.08, *p*=.776).						
	-FBS: not significant (F=3.25, *p*=.075)						
	-PP1: not significant (F=0.48, *p*=.489)						
Ko (2014) [18]	· Glycemic control	-	· Depression: reduced in the Exp (F=4.27, *p*=.043)	-	-	· Self-care behavior: increased in the Exp (t=-3.25, *p*=.002)	· Knowledge of GDM: not significant (t=–1.98, *p*=.052)
	-FBS: reduced in the Exp (t=5.03, *p*<.001).						
	-HbA1c: reduced in the Exp (t=3.94, *p*<.001)						
Kim (2013) [9]	· Glycemic control	-	-	-	· Maternal identity: increased in the Exp (U=–4.48, *p*<.001)	· Self-management: increased in the Exp (U=–3.80, *p*<.001)	-
	-SMBG (PP2): reduced in the Exp (U=–2.43, *p*=.015).						
	-HbA1c: not significant (U=–1.17, *p*=.238)						
Baek (2013) [16]	-	-	· Depression: reduced in the Exp (t=3.53, *p*=.001)	· Self-efficacy: increased in the Exp (t=–2.06, *p*=.047)	-	-	-
			· Anxiety: reduced in the Exp (t=5.49, *p*<.001)				
Park (2001) [21]	· Glycemic control	· Infant birth weight	-	-	-	-	-
	-SMBG	· Newborn sex					
	-HbA1c	· Perinatal complication					
	-Biochemical characteristics						
	· Gestational age						
	· Experience of insulin treatments						
	· Urinary ketones						
	· Delivery type						

Exp: experimental group, FBS: fasting blood sugar, GDM: gestational diabetes mellitus, HbA1c: glycosylated hemoglobin, OGTT: oral glucose tolerance test, PP1: post prandial 1-hour blood glucose test, PP2: post prandial 2-hour blood glucose test, SMBG: self-monitoring of blood glucose, T2DM: type 2 diabetes mellitus.
